# NF-κB-dependent miR-31/155 biogenesis is essential for TNF-α-induced impairment of endothelial progenitor cell function

**DOI:** 10.1038/s12276-020-0478-x

**Published:** 2020-08-07

**Authors:** Ji-Hee Kim, Ji-Yoon Kim, Minsik Park, Suji Kim, Taesam Kim, Joohwan Kim, Seunghwan Choi, Wonjin Park, Jong Yun Hwang, Jongseon Choe, Kwon-Soo Ha, Moo-Ho Won, Sungwoo Ryoo, Young-Guen Kwon, Young-Myeong Kim

**Affiliations:** 1 Department of Molecular and Cellular Biochemistry, Kangwon National University School of Medicine, Chuncheon, Gangwon-do 24341 South Korea; 2 Department of Anesthesiology and Pain Medicine, Hanyang University Hospital, Seoul, 04763 South Korea; 3 Department of Obstetrics and Gynecology, Kangwon National University School of Medicine, Chuncheon, Gangwon-do 24341 South Korea; 4 Department of Immunology, Kangwon National University School of Medicine, Chuncheon, Gangwon-do 24341 South Korea; 5 Department of Neurobiology, Kangwon National University School of Medicine, Chuncheon, Gangwon-do 24341 South Korea; 6 Department of Life Sciences, College of Natural Sciences, Kangwon National University, Chuncheon, Gangwon-do South Korea; 7 Department of Biochemistry, College of Life Science and Biotechnology, Yonsei University, Seoul, 03722 South Korea

**Keywords:** Differentiation, Cell biology

## Abstract

Endothelial progenitor cell (EPC) dysfunction impairs vascular function and remodeling in inflammation-associated diseases, including preeclampsia. However, the underlying mechanism of this inflammation-induced dysfunction remains unclear. In the present study, we found increases in TNF-α and miR-31/155 levels and reduced numbers of circulating EPCs in patients with preeclampsia. Patient-derived mononuclear cells (MNCs) cultured in autologous serum had decreased endothelial nitric oxide synthase (eNOS) expression, nitric oxide production, and differentiation into EPCs with angiogenic potential, and these effects were inhibited by a TNF-α-neutralizing antibody and miR-31/155 inhibitors. Moreover, TNF-α treatment of normal MNCs increased miR-31/155 biogenesis, decreased eNOS expression, reduced EPC differentiation, and impaired angiogenic potential. The TNF-α-induced impairment of EPC differentiation and function was rescued by NF-κB p65 knockdown or miR-31/155 inhibitors. In addition, treatment of MNCs with synthetic miR-31/155 or an eNOS inhibitor mimicked the inhibitory effects of TNF-α on eNOS expression and EPC functions. Moreover, transplantation of EPCs that had been differentiated from TNF-α-treated MNCs decreased neovascularization and blood perfusion in ischemic mouse hindlimbs compared with those of normally differentiated EPCs. These findings suggest that NF-κB activation is required for TNF-α-induced impairment of EPC mobilization, differentiation, and function via miR-31/155 biogenesis and eNOS downregulation. Our data provide a new role for NF-κB-dependent miR-31/155 in EPC dysfunction under the pathogenic conditions of inflammation-associated vascular diseases, including preeclampsia.

## Inflammatory vascular disease: miRNAs in blood vessel damage

miRNA molecules that inhibit the activity of specific genes are implicated in a cellular control network involved in some of the damaging effects of inflammation on blood vessels. Researchers in South Korea led by Young-Myeong Kim at Kangwon National University School of Medicine, Chuncheon, identified the link by studying cells from patients with the inflammatory condition pre-eclampsia, characterized by hypertension. They found that two miRNAs, miR-31 and miR-155, are involved in molecular signaling processes that impair the production of endothelial progenitor cells lining blood vessels, which is essential for maintenance and repair. The research also identified the key protein eNOS involved in the miRNA molecules’ mechanism of action. Understanding these miRNAs and the protein involved in their production and action may help researchers develop new treatments for blood vessel diseases associated with inflammation.

## Introduction

Angiogenesis and reendothelialization are key processes that regulate vascular function and homeostasis in physiological and pathological conditions^1^. Angiogenic dysregulation is associated with vascular malformation and contributes to the pathogenesis of many human diseases, such as cardiovascular disease, stroke, and preeclampsia^2,3^.

Angiogenesis and vascular function are dependent on endothelial cell function and are significantly modulated by circulating levels of endothelial progenitor cells (EPCs), which are capable of differentiating into mature endothelial cells^1,4^. Circulating EPC levels are controlled by differentiation and mobilization from the bone marrow in response to stromal-derived factor 1 (SDF-1), vascular endothelial growth factor (VEGF), and estrogen, which are known to stimulate endothelial nitric oxide synthase (eNOS) expression or activation in an Akt-dependent manner^5,6^. However, neither SDF-1, VEGF, nor estrogen is able to promote EPC mobilization in eNOS-deficient mice or in mice treated with an eNOS inhibitor, and both groups of mice show impaired angiogenesis related to EPC deficits^6–8^. Inhibition of eNOS-derived NO resulted in impaired differentiation of mononuclear cells (MNCs) into EPCs^9^. These findings indicate that the eNOS/NO pathway plays an important role in the regulation of EPC functions.

In contrast to activators of the eNOS/NO pathway, inflammatory cytokines, such as tumor necrosis factor-α (TNF-α) and interleukin-1β (IL-1β), have been shown to suppress EPC functions^10^. Decreased EPC mobilization and differentiation have also been observed in chronic inflammatory diseases, including preeclampsia and rheumatoid arthritis^5,11^, and these effects can be rescued by treatment with the anti-inflammatory drug dexamethasone and the anti-TNF-α antibody infliximab^11^. This finding suggests that inflammatory cytokines cause vascular dysfunction by impairing EPC functions in several clinical settings. Several studies have demonstrated that TNF-α and IL-1β downregulate eNOS expression via NF-κB-dependent biogenesis of miR-31/155^12–15^. However, no clear evidence regarding the role of NF-κB-dependent miR-31/155 in EPC differentiation and function has been reported.

MiRNAs negatively regulate target gene expression and contribute to impaired endothelial cell functions, resulting in vascular dysfunction^12–15^. Several miRNAs, including miR-221/222, miR-126, and miR-378, have been identified as regulating EPC differentiation and function by repressing selected mRNAs that control cell fate and behavior^16–18^. Despite this, little is known about inflammatory miRNAs that regulate EPC functions by targeting eNOS. Our findings demonstrate that TNF-α inhibits EPC differentiation and function via NF-κB-dependent biogenesis of miR-31/155, which target eNOS in preeclamptic conditions. We further provide new evidence that NF-κB can inhibit EPC mobilization and differentiation.

## Materials and methods

### Human specimens and animal experiments

Human specimens were obtained from 12-healthy pregnant women and 12 patients with preeclampsia according to protocols approved by the Institutional Review Board at Kangwon National University Hospital (KNUH-2017-01-010-004). Preeclampsia was defined by clinical diagnosis with systolic blood pressure ≥Nat mmHg or diastolic blood pressure ≥ 90 mmHg and proteinuria ≥0.3 g/24 h. Informed consent was obtained from all participants. This study conformed to the principles outlined in the Declaration of Helsinki. All animal experiments were performed in accordance with the guidelines of the Institutional Animal Care and Use Ethics Committee of Kangwon National University (KW-171228-1).

### Isolation, culture, and treatment of EPCs

MNCs were isolated from human whole venous blood and cord blood by conventional Ficoll-Hypaque density gradient centrifugation and resuspended in EGM-2 SingleQuots (#CC-4176, Lonza, Basel, Switzerland). Peripheral blood or cord blood MNCs (PBMNCs or CBMNCs, respectively, 4 × 10^6^ cells/mL) were seeded on a fibronectin (50 μg/mL; Sigma-Aldrich)-coated plate and incubated in a 5% CO_2_ incubator at 37 °C for 4 days. After being carefully washed, adherent MNCs were transfected with or without AllStars Negative Control for siRNA and miRNA mimic experiments (#1027280, Qiagen), miScript Inhibitor Negative Control for miRNA inhibitor experiments (#1027272, Qiagen), miRNA inhibitors (80 nM, Qiagen), miRNA mimics (80 nM, Qiagen), and NF-κB p65 siRNA (80 nM, Santa Cruz) as described previously^12–15^, followed by treatment with or without human TNF-α (10 ng/mL, R&D Systems) for another 4 days. In addition, some adherent MNCs were cultured in medium 199 supplemented with 30% human autologous serum in the presence or absence of an anti-human TNF-α antibody (5 μg/mL; #MAB210, R&D Systems) for another 4 days.

### Analysis of EPC differentiation

Cells were incubated with 10 μg/mL 1,1L were incubated with 10 on 30% human autologous serumadhe acetylated LDL (Dil-ac-LDL, Molecular Probes; Eugene, OR) in a CO_2_ incubator at 37 °C for 2 h. The cells were washed twice, fixed in 2% paraformaldehyde, and incubated with 10 μg/mL FITC-labeled *Ulex europaeus* agglutinin-1 (FITC-UEA-1, Sigma-Aldrich) for 2 h at room temperature. Images were obtained using a laser scanning confocal microscope. Cells that stained positively for both markers were considered EPCs. The number of EPCs per well was determined by counting four randomly selected high-power fields, and the fluorescence intensities were quantitated by fluorescence microscopy. In addition, cells were detached with a nonenzymatic cell dissociation solution (#C5789, Sigma-Aldrich) to avoid destruction of cell membrane markers. Cells were incubated with phycoerythrin (PE)-conjugated antibodies (BD PharMingen, San Jose, CA) against CD31 (#560983), CD34 (#555822), VEGF receptor-2 (KDR, #560872), and VE-cadherin (#560410) at 4 °C for 30 min. Cells were also incubated with an anti-von Willebrand factor (vWF) (#ab154193, Abcam, Cambridge, UK) and stained with FITC-conjugated goat anti-rabbit IgG, as described previously^19^. After being washed, the cells were fixed in 2% paraformaldehyde. A negative control with an isotype-matched antibody was included in each run. The expression levels of target proteins were determined by flow cytometry (FACSCalibur, BD).

### Circulating EPC counts

Circulating EPCs were detected by flow cytometry as described previously^20^. Anticoagulated peripheral blood (200 μL) was incubated for 30 min in the dark with a fluorescein isothiocyanate (FITC)-conjugated human CD34 antibody (Clone 581; BD Biosciences) and a PE-conjugated human KDR antibody (#560872, BD Biosciences). To assess the background, isotype controls were used as negative controls based on the species and immunoglobulin G subclass of each antibody. Erythrocytes were lysed with 2 mL of PharM Lyse solution (BD Pharmingen) for 7 min at room temperature, and the remaining cells were washed with phosphate-buffered saline (PBS) containing 0.5% bovine serum albumin. The cells were suspended in 400 μL of 2% paraformaldehyde and analyzed on a flow cytometer. The number of circulating EPCs was determined by the ratio of CD34^+^KDR^+^ cells per 100 PBMNCs.

### In vitro and in vivo angiogenesis assays

To measure in vitro angiogenesis, the migration and tube-like structure formation of EPCs were analyzed using Boyden chambers and growth factor-reduced Matrigel, respectively, as described previously^13^. In addition, a Matrigel plug angiogenesis assay was performed to assess the in vivo angiogenic activity of EPCs. Matrigel (0.3 mL) was mixed with vascular endothelial growth factor-A (VEGF-A, 10 ng/mL) and/or EPCs (1 × 10^6^ cells) suspended in PBS and subcutaneously implanted into the flanks of 7-week-old male athymic nude mice (Orient Bio Inc., Seongnam, South Korea). After 8 days, the animals were sacrificed, and the intact Matrigel plugs were carefully removed and visualized by phase contrast microscopy. The hemoglobin content of the plugs was determined using a Drabkin’s reagent kit (Sigma-Aldrich) according to the manufacturer’s instructions.

### Biochemical analysis

Total miRNAs were isolated from cells and plasma using the miRNeasy Mini Kit or the miRNeasy Serum/Plasma Kit (Qiagen, Hilden, Germany), and the levels of miR-31 and miR-155 were analyzed by qRT-PCR as described previously^13^. Total RNA was isolated from cultured cells using TRIzol reagent (Invitrogen, Carlsbad, CA), and the eNOS mRNA levels were quantified as described previously^13^. Intracellular NO levels were measured in situ in endothelial cells using DAF-FM diacetate as described previously^12^. Western blot analysis was performed as described previously^13^. The serum level of TNF-α was determined using the human Quantikine ELISA kit (#DTA00D, R&D Systems).

### Hindlimb ischemia model

Unilateral femoral artery occlusion was performed in 7-week-old male nude mice as described previously^21^. Putative EPCs were differentiated from CBMNCs and labeled with CM-Dil (#C7000, Invitrogen Molecular Probes) for 1 h at 37 °C. PBS-washed putative EPCs (5 × 10^5^ cells) were injected intramuscularly at four different locations in ischemic thigh muscles using 26-gauge needles. Sham-operated control animals were subjected to the same surgical protocol, but the femoral artery was not ligated. Blood flow in both hindlimbs was determined using laser-Doppler perfusion imaging (Moor Instruments, UK) for 21 h. The flow ratio of the occluded/nonoccluded leg was compared between experimental groups.

### Histochemical analysis

Mice were euthanized, and hindlimbs were harvested to analyze neovascularization at day 14 following transplantation of CM-Dil-labeled EPCs. Skeletal muscles were harvested from the of ischemic limbs and embedded in OCT compound for cryosectioning. To measure the capillary density and EPC incorporation, multiple 6 µm cryosections were stained with FITC-isolectin B4. Images of microvessels (green) and incorporated human EPCs (red) were visualized using a confocal microscope (Olympus, Tokyo, Japan).

### Statistical analysis

All data are presented as the mean ± SEM. All experiments were replicated at least three times, and representative results are shown. Statistical significance was evaluated using the unpaired Student’s *t*-test for comparison between two means. Multiple comparisons between ≥3 groups were performed by ANOVA, followed by Bonferroni’s multiple comparison test. *P*-values < 0.05 were considered significant.

## Results

### Elevated TNF-α and miR-31/155 levels in the serum and PBMNCs of preeclamptic women

**Fig. 1:**
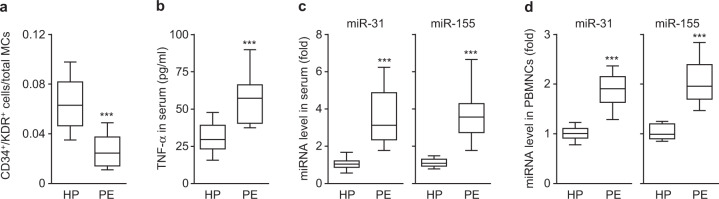
EPC, TNF-α, miR-31, and miR-155 levels in healthy pregnant women and preeclamptic patients. **a** The levels of circulating CD34^+^/KDR^+^ EPCs in peripheral blood from healthy pregnant (HP) and preeclamptic women (PE). **b**, **c** The levels of TNF-α, miR-31, and miR-155 in serum from healthy controls and preeclamptic women. **d** The levels of miR-31 and miR-155 in PBMNCs from healthy and preeclamptic subjects. *n* = 12 per group, ***P < 0.001.

We first examined the levels of circulating EPCs and the levels of TNF-α and miR-31/155 in the serum and PBMNCs of healthy pregnant and preeclamptic women. As shown in previous studies^20,22^, circulating levels of EPCs were significantly decreased in preeclamptic patients compared with healthy individuals (Fig. 1a); however, the plasma levels of TNF-α and miR-31/155 were markedly elevated in preeclamptic patients (Fig. 1b and c). Similarly, miR-31/155 levels were also increased in PBMNCs from patients compared with those from healthy subjects (Fig. 1d). These results suggest that TNF-α and miR-31/155 are involved in negatively regulating the mobilization and differentiation of EPCs in patients with preeclampsia.

### Patient autologous serum-mediated suppression of EPC differentiation is mediated by TNF-α and miR-31/155

**Fig. 2:**
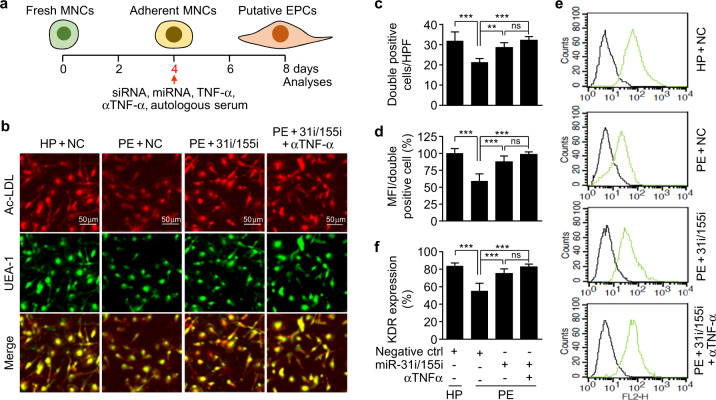
Patient autologous serum-mediated suppression of EPC differentiation is prevented by inhibitors of TNF-α and miR-31/155. **a** Diagram depicting the protocol of EPC differentiation from MNCs and the treatment schedule. **b** Adherent PBMNCs from healthy pregnant (HP) and preeclamptic women (PE) were transfected with miRNA inhibitor negative control (NC) or miR-31/155 inhibitors (miR-31i/155i) and differentiated into EPCs in autologous serum-supplemented culture media with or without a human TNF-α-neutralizing antibody (αTNF-α). EPCs were identified by confocal microscopy after staining with Dil-ac-LDL and FITC-UEA-1. **c** The number of double-positive (yellow) cells per high-power field (HPF) was counted. **d** The mean fluorescence intensity (MFI) of double-positive cells was quantitated for each cell using ImageJ software. **e**, **f** The expression levels of KDR were quantitated by flow cytometry. *P* < 0.05, **P < 0.01, and ***P < 0.001. ns not significant.

To examine the role of TNF-α and miR-31/155 in EPC dysfunction in preeclamptic patients, PBMNCs were isolated from healthy and preeclamptic subjects, transfected with or without miR-31/155 inhibitors, and cultured in autologous serum in the presence or absence of a human TNF-α-neutralizing antibody as an in vivo model of preeclampsia (Fig. 2a). PBMNCs isolated from preeclamptic patients exhibited decreased differentiation into EPCs, as determined by counting double-positive cells stained with Dil-ac-LDL and FITC-UEA-1, which is a typical characteristic of EPCs, compared with those from healthy subjects (Fig. 2b, c). Double-positive fluorescence intensity was also decreased in cells from patients compared with cells from healthy individuals (Fig. 2b, d). In addition, the double-positive cells showed early EPC-like morphology with a higher degree of spindle elongation in healthy controls than in preeclamptic patients (Fig. 2b). The decreased differentiation of EPCs from patient-derived MNCs was significantly recovered by treatment with miR-31/155 inhibitors and was further improved by cotreatment with an anti-TNF-α antibody, but the difference was not statistically significant (Fig. 2b–d). Notably, the expression of the endothelial cell-specific surface marker KDR was lower in patient PBMNC-derived EPCs than in healthy individual-derived cells, and the decreased KDR levels were significantly restored by treatment with miR-31/155 inhibitors and further improved slightly by cotreatment with an anti-TNF-α antibody (Fig. 2e, f). These results suggest that TNF-α and miR-31/155 act as negative regulators of EPC differentiation in patients with preeclampsia.

### Patient serum-derived TNF-α and miR-31/155 suppress eNOS expression and EPC functions

**Fig. 3:**
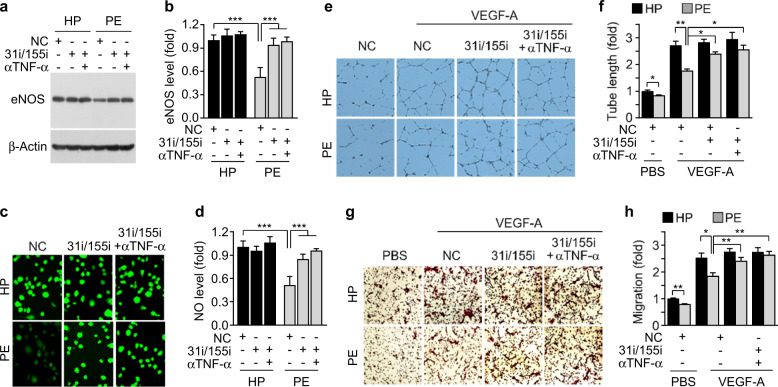
Patient autologous serum suppresses eNOS and EPC functions. Adherent PBMNCs from healthy pregnant (HP) and preeclamptic women (PE) were transfected with miRNA inhibitor negative control (NC) or miR-31/155 inhibitors (31i/155i) and differentiated into EPCs in autologous serum-supplemented culture media in the presence or absence of a human TNF-α-neutralizing antibody (αTNF-α). **a**, **b** The expression levels of eNOS were analyzed by western blotting and quantitated using ImageJ. **c**, **d** Intracellular NO levels were analyzed by confocal microscopy and quantitated by ImageJ software. **e**, **f** EPCs were stimulated with VEGF-A on growth factor-reduced Matrigel, and tube formation was photographed using an inverted microscope and quantitated using ImageJ software. **g**, **h** EPC migration in response to VEGF-A was determined by the Boyden chamber assay and quantitated using ImageJ software. *n* = 4. *P < 0.05, **P < 0.01, and ***P < 0.001.

Because miR-31/155 were shown to negatively regulate the eNOS/NO pathway, which is crucially implicated in EPC functions^6^, we investigated whether patient autologous serum suppresses the eNOS/NO axis and the in vitro angiogenic activity of PBMNC-derived EPCs. eNOS expression and NO production were significantly decreased in EPCs differentiated from patient-derived PBMNCs cultured in autologous serum compared with those of cells treated with serum from healthy pregnant women. This decrease was recovered by treatment with miR-31/155 inhibitors or in combination with a TNF-α-neutralizing antibody (Fig. 3a–d). Moreover, patient-derived EPCs showed a significant decrease in angiogenic properties, such as tube formation and migration, in response to VEGF-A compared with those of healthy EPCs, and the decreased angiogenic responses of patient-derived EPCs were significantly rescued by treatment with miR-31/155 inhibitors and further increased slightly by cotreatment with a TNF-α-neutralizing antibody (Fig. 3e–h). These results suggest that TNF-α and miR-31/155 are responsible for EPC dysfunction in preeclamptic patients through downregulation of eNOS.

### NF-κB is essential for TNF-α-induced eNOS downregulation during EPC differentiation by inducing miR-31/155

**Fig. 4:**
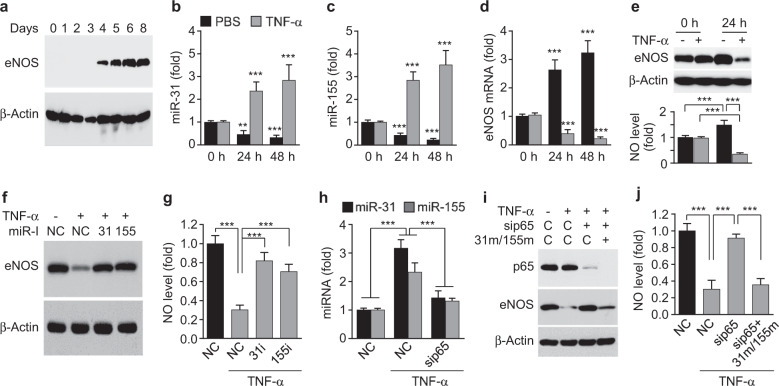
TNF-α inhibits eNOS expression via NF-κB-dependent induction of miR-31/155. **a** The expression pattern of eNOS during the differentiation of CBMNCs into EPCs. **b**–**e** CBMNCs were stimulated with or without TNF-α (10 ng/ml) for the indicated time periods. Quantitative levels of miR-31 (**b**), miR-155 (**c**), eNOS mRNA (**d**), and eNOS protein and NO production (**e**) were determined by qRT-PCR, western blotting, and confocal microscopy (*n* = 4). **P < 0.01 and ***P < 0.001 *vs*. the initial control level. **f**, **g** CBMNCs were transfected with miRNA inhibitor negative control (NC) or miRNA inhibitors (miR-I; 31i, miR-31 inhibitor and 155i, miR-155 inhibitor), followed by treatment with TNF-α for 2 days. The levels of eNOS protein and NO production were determined by western blotting and confocal microscopy, respectively (*n* = 4). ***P < 0.001. **h**–**j** CBMNCs were transfected with AllStars negative control (NC or C), NF-κB p65 siRNA (sip65), or miR-31/155 mimics (31 m/155 m), followed by stimulation with TNF-α for 24 h. **h**–**j** The levels of miR-31 and miR-155 (**h**), eNOS protein (**i**), and NO production (**j**) were analyzed (*n* = 4). ***P < 0.001.

We next examined the roles of TNF-α and miR-31/155 in eNOS expression during the differentiation of CBMNCs into EPCs. The expression of eNOS occurred in healthy CBMNCs on day 4 of culture and increased until day 6 (Fig. 4a). Treatment of CBMNCs with TNF-α resulted in a significant increase in miR-31/155 levels with subsequent reductions in eNOS expression and NO production in a time-dependent manner, whereas untreated control cells gradually suppressed expression levels of both miRNAs and upregulated the eNOS/NO pathway (Fig. 4b–e). Notably, the TNF-α-induced decreases in eNOS and NO levels were prevented by transfection with a miR-31 or miR-155 inhibitor (Fig. 4f, g). In addition, the TNF-α-induced increase in miR-31/155 biogenesis was markedly blocked by knockdown of NF-κB p65, resulting in the restoration of TNF-α-induced suppression of eNOS expression and NO production (Fig. 4h–j). The recovery effects of NF-κB p65 knockdown were abrogated by transfection with miR-31/155 mimics (Fig. 4h–j). These results suggest that NF-κB-dependent miR-31/155 are essential for TNF-α-induced impairment of the eNOS/NO pathway during the differentiation of MNCs into EPCs.

### TNF-α inhibits the differentiation of MNCs into EPCs via miR-31/155 biogenesis

**Fig. 5:**
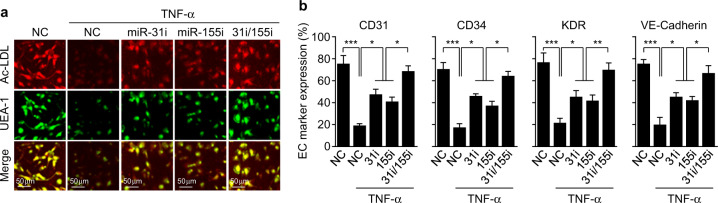
TNF-α inhibits the differentiation of MNCs into EPCs via miR-31/155 biogenesis. CBMNCs were transfected with negative control for miRNA inhibitor (NC), miR-31 inhibitor (miR-31i or 31i), miR-155 inhibitor (miR-155i or 155i), or miR-31/155 inhibitors (31i/155i), followed by differentiation into EPCs in the presence or absence of TNF-α. **a** The differentiation of CBMNCs to EPCs was determined by double-positive staining with Dil-ac-LDL and FITC-UEA-1. **b** Cells were labeled with antibodies against EPC marker proteins and analyzed by flow cytometry (*n* = 3). *P < 0.05, **P < 0.01, and ***P < 0.001.

To investigate the role of miR-31/155 in TNF-α-mediated suppression of EPC differentiation, we treated CBMNCs with TNF-α after transfection with miR-31/155 inhibitors and assessed the differentiation of these cells into EPCs by analyzing DiI-Ac-LDL uptake, FITC-UEA-1 binding, and EPC-specific marker expression. CBMNCs treated with TNF-α had decreased cellular uptake of DiI-Ac-LDL and binding with FITC-UEA-1. These decreases were rescued by transfection with a miR-31 or miR-155 inhibitor and synergistically recovered by the combination of both inhibitors (Fig. 5a). Similarly, TNF-α also markedly inhibited the expression of EPC-specific surface markers, including CD31, CD34, KDR, and VE-cadherin, in CBMNCs. The inhibitory effects of TNF-α were prevented by transfection with a miR-31 or miR-155 inhibitor and further abrogated by the combination of both inhibitors (Fig. 5b). These results suggest that miR-31/155 play a crucial role in TNF-α-mediated inhibition of EPC differentiation.

### TNF-α impairs EPC differentiation and function via NF-κB-mediated miR-31/155 biogenesis

**Fig. 6:**
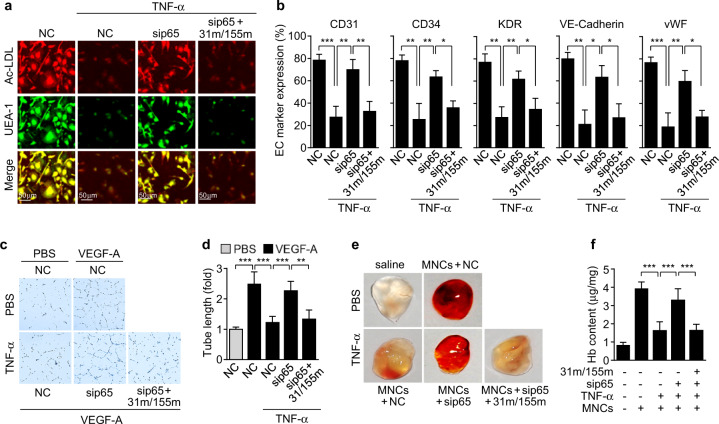
TNF-α inhibits EPC differentiation via NF-κB-dependent biogenesis of miR-31/155. CBMNCs (MNCs) were transfected with negative control (NC), NF-κB p65 siRNA (sip65), or miR-31/155 mimics (31 m/155 m), followed by treatment with or without TNF-α. **a** Representative images of EPCs characterized by double-positive staining with Dil-ac-LDL and FITC-UEA-1. **b** Cells were labeled with antibodies against EPC marker proteins and analyzed by flow cytometry (*n* = 3). **c**, **d** Tube formation of EPCs in response to VEGF-A was photographed using an inverted microscope and quantitated using ImageJ software (*n* = 4). **e**, **f** Matrigel containing low-dose VEGF-A (5 ng/ml) was mixed with or without 2 × 10^5^ EPCs differentiated from CBMNCs and subcutaneously injected into athymic nude mice. **e** After 8 days, the Matrigel plugs were harvested and photographed. **f** The hemoglobin (Hb) content in Matrigel plugs was determined using Drabkin’s reagent (*n* = 4). *P < 0.05, **P < 0.01, and ***P < 0.001.

We investigated the role of NF-κB-dependent miR-31/155 in TNF-α-mediated suppression of EPC differentiation. Treatment of CBMNCs with TNF-α resulted in marked inhibition of EPC differentiation, as determined by Dil-ac-LDL uptake and FITC-UEA-1 binding, and this inhibition was blocked by siRNA-mediated knockdown of NF-κB p65 (Fig. 6a). Notably, the beneficial effect of p65 knockdown disappeared upon transfection with miR-31/155 mimics (Fig. 6a). We next examined the role of NF-κB-dependent miR-31/155 in the expression of EPC surface markers on CBMNCs treated with TNF-α using FACS analysis. TNF-α treatment suppressed the expression of EPC-specific markers, such as CD31, CD34, VE-cadherin, KDR, and vWF, compared with those in untreated control cells, and these suppressive effects were largely recovered by p65 knockdown (Fig. 6b). Moreover, the recovery effects of p65 knockdown were nullified by miR-31/155 mimics (Fig. 6b). We further evaluated the effect of TNF-α-induced miR-31/155 on the functional differentiation of EPCs by analyzing their angiogenic potential in vitro and in vivo. TNF-α pretreatment inhibited the in vitro tube-forming activity of CBMNC-derived EPCs in response to VEGF-A, and this inhibitory effect was markedly rescued by p65 knockdown (Fig. 6c and d). We also performed a Matrigel plug assay to evaluate the angiogenic activity of EPCs in athymic nude mice in vivo. Matrigel plugs mixed with low-dose VEGF-A [5 ng/mL, one twentieth of normal dose^23^] exhibited a very pale pink color, whereas the plugs containing normally differentiated EPCs showed a strong red color, indicating an abundance of erythrocytes within the lumen as a result of functional neovascularization (Fig. 6e). This angiogenic effect was markedly suppressed by the replacement of the EPCs with TNF-α-treated CBMNC-derived EPCs, resulting in light red- or straw-yellow-colored Matrigel plugs. The suppressive effect of TNF-α was largely prevented in EPCs that were differentiated from p65-knockdown CBMNCs; however, the recovery effects of p65 knockdown disappeared upon cotransfection with miR-31/155 mimics (Fig. 6e). Similar results were also observed by measuring the hemoglobin content in the gel plugs (Fig. 6f). These data suggest that TNF-α impairs EPC differentiation and function via NF-κB-dependent miR-31/155 biogenesis.

### Synthetic miR-31/155 and an eNOS inhibitor suppress EPC differentiation

**Fig. 7:**
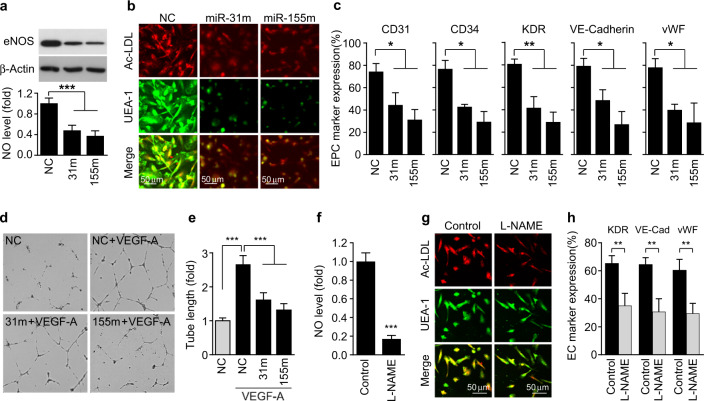
miR-31/155 mimics and an eNOS inhibitor impair EPC differentiation. **a**–**e** CBMNCs were transfected with negative control (NC), miR-31 mimic (miR-31m or 31 m), or miR-155 mimic (miR-155m or 155 m) and differentiated into EPCs in fresh media. **a** The levels of eNOS protein and NO production were determined by western blotting and confocal microscopy (*n* = 4). **b** Representative images of EPCs characterized by double-positive staining with Dil-ac-LDL and FITC-UEA-1. **c** The expression levels of EPC markers were analyzed by flow cytometry (*n* = 3). **d**, **e** The angiogenic potential of EPCs was determined by a tube formation assay (*n* = 4). **f**–**h** Adherent CBMNCs were cultured with or without L-NAME (1 mM) for 4 days. **f** Intracellular NO production was determined by confocal microscopy (*n* = 4). **g** EPC differentiation was confirmed by staining with Dil-ac-LDL and FITC-UEA-1. **h** The expression levels of EPC markers were analyzed by flow cytometry (*n* = 3). *P < 0.05, **P < 0.01, and ***P < 0.001.

We investigated whether synthetic miR-31/155 mimics regulate the differentiation of CBMNCs into EPCs. Transfection of CBMNCs with a miR-31 or miR-155 mimic suppressed eNOS expression and NO production (Fig. 7a) and prevented EPC differentiation, as confirmed by costaining with Dil-ac-LDL and FITC-UEA-1 (Fig. 7b). Moreover, FACS analysis showed that transfection of CBMNCs with a miR-31 or miR-155 mimic downregulated EPC markers, such as CD31, CD34, KDR, VE-cadherin, and vWF, compared with those of the untreated control (Fig. 7c), as shown in the aforementioned results obtained from TNF-α-treated cells (Fig. 6b). Both synthetic miRNA mimics inhibited the tube-forming activity of EPCs in response to VEGF-A (Fig. 7d, e). In addition, the eNOS inhibitor N^G^-nitro-L-arginine methyl ester (L-NAME) suppressed NO production and EPC differentiation, as determined by fluorescence microscopy and FACS analysis (Fig. 7f–h). These data suggest that miR-31/155 directly inhibit EPC differentiation by inhibiting the eNOS/NO axis.

### NF-κB-dependent miR-31/155 impair EPC abilities to improve therapeutic neovascularization

**Fig. 8:**
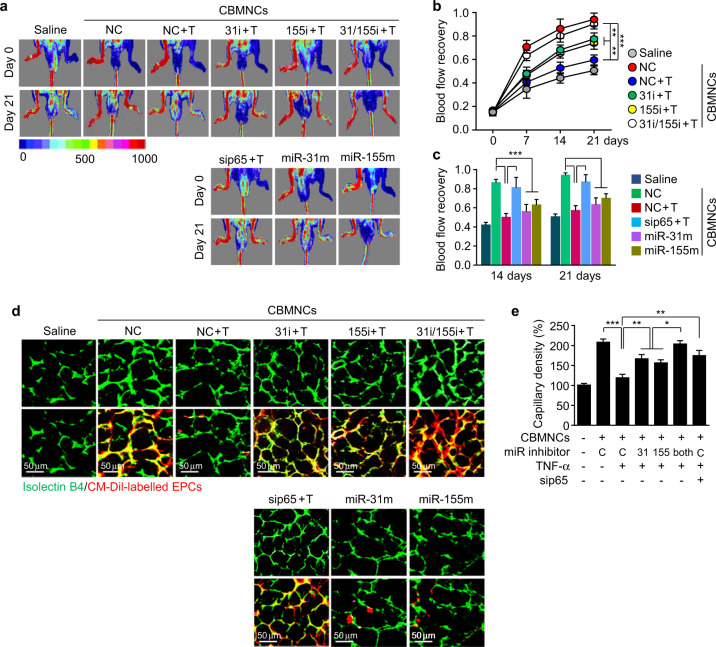
EPCs differentiated from CBMNCs that were treated with TNF-α or miR-31/155 mimics lose their capability to improve therapeutic neovascularization in mouse ischemic hindlimbs. CBMNCs were transfected with negative control (NC or C), NF-κB p65 siRNA (sip65), miR-31 inhibitor (miR-31i), miR-155 inhibitor (155i), miR-31 mimic (miR-31m), or miR-155 mimic (miR-155m) and differentiated into EPCs in fresh media with or without TNF-α (T). Putative EPCs were intramuscularly transplanted into the ischemic hindlimbs of athymic nude mice. **a** Representative laser-Doppler perfusion image of mouse hindlimbs at days 0 and 21 after hindlimb ischemia**. b**, **c** Time course of computer-assisted analysis of the ratio of blood flow between ischemic and nonischemic contralateral limbs (*n* = 5). **d** Representative images of capillaries (FITC-isolectin B4, green) and incorporated EPCs (yellow and red) in transverse sections of skeletal muscles from ischemic mouse hindlimbs that received CM-Dil-labeled putative EPCs. **e** Quantitative analysis of capillary density (*n* = 5). *P < 0.05, **P < 0.01, and ***P < 0.001.

We assessed the therapeutic angiogenic capacity of EPCs differentiated from CBMNCs that were treated with or without TNF-α in a mouse model of hindlimb ischemia using laser-Doppler perfusion imaging. Intramuscular injection of normally differentiated EPCs markedly improved blood flow in the ischemic limbs when compared with that of saline-treated controls, whereas EPCs differentiated from CBMNCs treated with TNF-α exhibited significantly inhibited abilities to facilitate blood flow recovery (Fig. 8a–c). The inhibitory effect of TNF-α was prevented by transfection with a miR-31 or miR-155 inhibitor, was effectively blocked by the combination of both miRNA inhibitors, and was markedly abolished by p65 knockdown (Fig. 8a–c). In addition, EPCs derived from CBMNCs transfected with a miR-31 or miR-155 mimic showed decreased abilities to recover blood flow in ischemic limbs (Fig. 8a, c). In addition, higher capillary densities were observed in the ischemic limbs of mice receiving normal EPCs than in those of saline-treated animals, and this effect was not observed in EPCs that were differentiated from CBMNCs treated with TNF-α. The inhibitory effect of TNF-α on neovascularization was mitigated by transfection with the miR-31 or miR-155 inhibitor, was synergistically prevented by the combination of both inhibitors, and was rescued by p65 knockdown (Fig. 8d, upper panel, e). Moreover, EPCs derived from CBMNCs transfected with a miR-31 or miR-155 mimic were restricted in their ability to facilitate neovascularization (Fig. 8d, upper panel). We further examined the role of TNF-α-mediated miR-31/155 biogenesis in EPC-induced neovascularization by assaying the incorporation of EPCs into neovessels. Normally differentiated EPCs were detected and largely incorporated into the newly formed microvessels in mouse ischemic limbs, and this incorporation was markedly reduced in the limbs of mice administered EPCs derived from CBMNCs treated with TNF-α (Fig. 8d, lower panel). The decreased incorporation of EPCs derived from TNF-α-treated CBMNCs was recovered by transfection with the miR-31 or miR-155 inhibitor, was further rescued by the combination of both inhibitors, and was reversed by p65 knockdown (Fig. 8d, lower panel). In addition, transfection with a miR-31 or miR-155 mimic prevented the incorporation of normally differentiated EPCs into new capillaries (Fig. 8d, lower panel). These results suggest that NF-κB-dependent miR-31/155 are essential for TNF-α-induced impairment of EPC differentiation and function to improve blood flow via neovascularization in ischemic tissues.

## Discussion

Inflammation may have a negative effect on EPCs. Excessive and chronic inflammatory responses result in reduced numbers of circulating EPCs^11,24^, leading to development of vascular dysfunction in inflammation-associated disorders, including cardiovascular disease, rheumatoid arthritis, and preeclampsia^11,20,25,26^. This finding suggests that inflammation is crucially involved in impaired EPC functions; however, the mechanism linking inflammation to EPC dysfunction has not been studied. The present study clearly demonstrates that TNF-α inhibits EPC differentiation and angiogenic potential by stimulating NF-κB-dependent biogenesis of miR-31/155, which downregulate the eNOS/NO pathway. This finding suggests that NF-κB plays an essential role in inflammation-associated vascular disease by impairing EPC differentiation and function. Thus, our findings provide a novel mechanism underlying impaired EPC differentiation in inflammation-associated diseases.

Preeclampsia, clinically characterized by hypertension and proteinuria^27^, has been recognized as an inflammatory disease associated with placenta-induced increases in inflammatory cytokines, such as TNF-α and IL-6^28^. Recent studies have demonstrated that inflammatory cytokines induce functional impairment of endothelial cells by inhibiting the eNOS/NO pathway^12–14^. In addition, increasing evidence shows that the number of EPCs was reduced in maternal blood and umbilical cord blood of patients with preeclampsia, leading to a defect in vascular angiogenesis and remodeling in maternal and fetal compartments^20,22,29,30^. However, the pathogenic mechanism underlying the reduction in circulating EPCs in women with preeclampsia remains unknown. Our results suggest that the inflammatory cytokine TNF-α plays an important role in impairing EPC mobilization and differentiation in patients.

In addition to preeclampsia, patients with other inflammation-related diseases, such as rheumatoid arthritis, atherosclerosis, obesity, and type 2 diabetes, exhibit reduced circulating levels of EPCs when compared with those of healthy individuals^11,25,31,32^. TNF-α is a central risk factor in the pathogenesis of these diseases, and treatment of rheumatoid arthritis with the anti-TNF-α antibody infliximab improves the number and functional properties of EPCs^11,24^, indicating a close relationship between TNF-α and EPC dysfunction. Moreover, when treated with the anti-inflammatory glucocorticoid dexamethasone, patients with rheumatoid arthritis showed significant increases in the levels of circulating EPCs, along with decreases in the serum levels of TNF-α and IL-6^11^, suggesting that inflammatory cytokines are important factors leading to EPC dysfunction. Consistently, we also found that treatment of MNCs with TNF-α or preeclamptic patient-derived autologous serum led to a significant decrease in EPC differentiation, an effect that was mostly reversed by a neutralizing TNF-α antibody, NF-κB p65 knockdown, or miR-31/155 inhibitors. This finding indicates that elevated levels of circulating TNF-α may decrease the number and function of circulating EPCs in patients with inflammatory vascular diseases.

EPCs have been shown to express eNOS, an established marker of endothelial cells, although eNOS expression in MNCs is debatable^4^. We found that eNOS expression gradually increased during the differentiation of MNCs into EPCs. The eNOS/NO pathway improves vasorelaxation, angiogenesis, and vascular remodeling by promoting EPC mobilization and differentiation^6,33^. Indeed, mice deficient in eNOS showed a reduction in VEGF-induced mobilization of EPCs from the bone marrow^6^. Treatment of MNCs either with the endogenous NOS inhibitor asymmetric dimethylarginine (ADMA) or the chemical NOS inhibitor L-NAME repressed the in vitro differentiation of MNCs into EPCs^34,35^, and the plasma concentration of ADMA was inversely correlated with the number of circulating EPCs and was related to the severity of coronary artery disease^34^. These results suggest that the decreased population of circulating EPCs in inflammatory diseases is due to downregulation of eNOS expression or NO production by elevated levels of proinflammatory cytokines, including TNF-α^11,13,14^. Our previous studies showed that TNF-α downregulated eNOS expression and NO production, leading to impaired endothelial and vascular functions^12–14^. Similarly, in this study, we found that TNF-α inhibited the differentiation of MNCs into EPCs by inhibiting eNOS expression and NO production.

Generally, eNOS is constitutively expressed in endothelial cells, and its catalytic activity is largely regulated by posttranslational modification^36^. However, eNOS expression can also be regulated at both the transcriptional and posttranscriptional levels in pathophysiological conditions. In fact, estrogen increases the transcriptional activity of the eNOS promoter, resulting in increased EPC mobilization and reendothelialization in injured vessels^8,37^. In contrast, inflammatory stimulants, including TNF-α and IL-1β, suppress eNOS expression by decreasing the stability of eNOS mRNA, leading to impaired endothelial cell functions^12–14^. Several studies have demonstrated that some miRNAs negatively regulate eNOS expression by targeting the eNOS mRNA transcript^12–14^. Among these miRNAs, miR-31/155 are upregulated in endothelial cells by inflammatory cytokines and inhibit the eNOS/NO axis, resulting in inhibition of vascular homeostasis^12–14^. Plasma levels of both miRNAs and TNF-α were found to be increased in patients with preeclampsia and rheumatoid arthritis^13,14,38^ and were inversely correlated with eNOS expression in patient-derived endothelial cells^15,16^. Consistent with these results, we found that TNF-α and miR-31/155 levels were increased in the serum and PBMNCs of preeclamptic patients, thereby suppressing the differentiation of MNCs into EPCs by downregulating eNOS. This finding indicates that TNF-α-dependent miR-31/155 inhibit EPC mobilization and differentiation by suppressing the eNOS/NO axis. Unfortunately, both miRNAs can target eNOS mRNA transcripts in humans and nonhuman primates but not other species, including mice^13,14^. However, our previous results showed that TNF-α decreases mouse eNOS expression in an NF-κB-dependent manner^13,14^, suggesting that TNF-α-induced unidentified miRNAs are involved in the downregulation of mouse eNOS. Thus, miR-31/155 may elicit EPC dysfunction in inflammatory diseases in a species-specific manner.

NF-κB plays a pivotal role in regulating diverse cellular processes, such as inflammation, apoptosis, and cell fate decisions. In addition, NF-κB impairs endothelial function under inflammatory conditions by upregulating miR-31/155, which inhibit the eNOS/NO pathway^12,13^. Because TNF-α is produced via NF-κB activation and in turn activates the NF-κB pathway, a positive feed-forward loop between TNF-α and NF-κB may contribute to the impairments in endothelial and EPC functions. Although TNF-α is responsible for impairing vascular function by reducing EPC mobilization and differentiation^11,24^, the role of NF-κB in impaired EPC differentiation and function is largely unknown. Here, we elucidated that NF-κB activation was crucially involved in TNF-α-induced impairment of EPC function by downregulating eNOS via miR-31/155 biogenesis. These results indicate that NF-κB is a determining factor in regulating EPC functions under chronic inflammation-associated pathological conditions, including preeclampsia. Thus, inhibiting the NF-κB pathway can significantly improve vascular function and remodeling in the pathogenic condition of preeclampsia by preventing endothelial cell dysfunction, as well as by restoring circulating EPC levels and functions.

In summary, the present study demonstrates that TNF-α plays a crucial role in the pathogenesis of preeclampsia, which is associated with decreased EPC differentiation and function, similar to that of endothelial dysfunction^12–14^, via NF-κB-mediated biogenesis of miR-31/155 and the subsequent inhibition of the eNOS/NO pathway. This finding indicates that NF-κB plays a crucial role in impairing vascular function and remodeling by suppressing EPC and endothelial cell functions in human chronic inflammatory diseases. These findings provide new insights into the role of NF-κB in EPC mobilization and differentiation. Therefore, targeting the NF-κB-miR-31/155 axis may be a promising therapeutic approach for inflammatory vascular diseases, including preeclampsia.
